# Supplementary material for: Determinants of Carotid Wall Echolucency in a Cohort of European High Cardiovascular Risk Subjects: A Cross-Sectional Analysis of IMPROVE Baseline Data
Source: Biomedicines. 2024 Mar 26;12(4):737. doi: 10.3390/biomedicines12040737 (PMC11154292; doi:10.3390/biomedicines12040737)
Supplement: Supplementary file 1 [file biomedicines-12-00737-s001.zip › biomedicines-2785992-supplementary.pdf]

SUPPLEMENTARY MATERIALS

Table S1. Characteristics of participants according to quartiles of carotid plaque-GSM of the darkest one between right and left carotid plaque.

Quartiles of carotid plaque-GSM						
	**More echolucent (darkest)**			**Less echolucent (brightest)**		
	**(<23.5) (n=534)**	**[23.5-29.1] (n=535)**	**(29.1-36.4) (n=535)**	**(>36.4) (n=534)**	**Std. Beta coefficients**	**P_{trend}**
Kuopio, No. (%)	189 (35.4)	148 (27.7)	71 (13.3)	29 (5.4)		
Stockholm, No. (%)	95 (17.8)	126 (23.6)	114 (21.3)	68 (12.7)		
Groningen, No. (%)	51 (9.6)	63 (11.8)	114 (21.3)	138 (25.8)	-2.89*	**<.0001**
Paris, No. (%)	132 (24.7)	81 (15.1)	51 (9.5)	30 (5.6)		
Milan, No. (%)	48 (9.0)	77 (14.4)	113 (21.1)	127 (23.8)		
Perugia, No. (%)	19 (3.6)	40 (7.5)	72 (13.5)	142 (26.6)		
**Anthropometric variables**						
Male sex, No. (%)	273 (51.1)	281 (52.5)	292 (54.6)	260 (48.7)	-0.14	0.59
Age (years)	65.5±5.4	65.5±5.2	64.7±5.2	63.9±5.5	-1.02	**<.0001**
Height (m)	1.67±0.1	1.68±0.1	1.69±0.1	1.68±0.1	0.03	0.62
Weight (kg)	77.3±14.4	77.8±15.8	78.1±14.5	76.3±16.3	-0.15	0.37
BMI (Kg/m^2)	27.5±4.1	27.3±4.4	27.3±4.0	27.0±4.5	-0.24	0.11
Waist (cm)	95.3±12.2	95.6±13.1	95.1±12.1	94.1±12.7	-0.31	0.08
Hip (cm)	102.3±9.2	102.7±9.8	102.9±9.0	102.3±10.1	0.11	0.82
Waist/hip ratio	0.93±0.1	0.93±0.1	0.92±0.1	0.92±0.1	-0.61	0.004
Heart rate (bpm)	64.8±8.9	66.1±9.0	66.5±9.5	67.3±9.0	1.08	**<.0001**
DBP (mmHg)	81.9±10.7	83.2±10.4	82.8±9.6	82.8±9.1	0.29	0.29
SBP (mmHg)	145.2±20.6	144.5±19.5	143.1±18.1	142.0±17.2	-0.5	0.003
Pulse Pressure (mmHg)	63.3±15.5	61.3±14.6	60.4±13.9	59.3±13.2	-0.86	**<.0001**
**Educational level (study years)**	10.7±3.8	10.6±3.7	10.5±4.0	10.1±4.0	-0.63	0.08
**Physical activity**						
Low, No. (%)	89 (16.8)	95 (17.9)	125 (23.5)	128 (24.0)		
Medium, No. (%)	239 (45.1)	236 (44.4)	237 (44.5)	266 (49.8)	-0.96	**<.0001**
High, No. (%)	202 (38.1)	200 (37.7)	170 (32.0)	140 (26.2)		
**Biochemical risk factors**						
Total Cholesterol (mg/dL)	210.5±44.1	211.8±41.9	212.4±44.4	212.9±40.9	0.10	0.35
HDL Cholesterol (mg/dL)	48.2±14.3	48.1±14.1	46.6±13.1	47.5±12.8	-0.21	0.15
Triglycerides (mg/dL)	119 (84; 170)	116 (83; 170)	119 (84; 172)	119 (84; 168)	0.13	0.83
LDL Cholesterol (mg/dL)	135.0±39.2	137.3±37.9	138.2±39.3	138.5±36.5	0.17	0.13
Uric acid (mg/dL)	5.3 (4.4; 6.0)	5.3 (4.5; 6.1)	5.3 (4.4; 6.2)	5.2 (4.3; 6.1)	-0.04	0.61
hs-CRP (mg/L)	1.7 (0.7; 3.4)	1.8 (0.8; 3.8)	2.1 (0.9; 3.8)	2.2 (0.9; 4.2)	0.54	0.01
Blood glucose (mmol/L)	5.8 (5.1; 6.8)	5.7 (5.2; 6.6)	5.5 (4.9; 6.3)	5.4 (4.8; 6.2)	-1.04	**<.0001**
Creatinine (μmol/L)	80.3 (69.9; 92.0)	78.5 (67.3; 89.9)	80.3 (69.5; 91.9)	80.2 (69.9; 91.3)	0.17	0.21
Adiponectin (μg/mL)	9.2 (4.7; 15.8)	10.0 (5.8; 16.0)	10.4 (6.5; 17.4)	11.6 (7.4; 17.3)	1.12	**<.0001**
**Hematological variables**						
Leucocytes (WBC) (x 10^9/L)	6.0 (5.1; 6.9)	5.9 (5.1; 7.0)	6.1 (5.2; 7.2)	6.1 (5.2; 7.1)	0.35	0.24
Erythrocytes (RBC) (x 10^{12}/L)	4.62±0.4	4.65±0.4	4.70±0.4	4.68±0.5	0.59	0.01
Haemoglobin (g/dL)	14.2±1.3	14.2±1.2	14.3±1.1	14.1±1.2	-0.19	0.46
Haematocrit (%)	41.9±3.9	42.2±3.9	42.3±3.1	42.2±4.5	0.32	0.15
MCV (fl)	90.8±4.6	90.7±4.6	90.1±5.1	90.1±5.7	-0.6	0.007
MCH (pg)	30.8±1.6	30.7±1.7	30.5±1.9	30.4±1.8	-0.95	**<.0001**
MCHC (g/dL)	33.9±1	33.9±1	33.8±1.2	33.7±1.1	-0.88	**0.00002**
Platelets (x 109/L)	235.4±58.2	239.4±56.0	237.2±58.6	242.4±57.1	0.38	0.10

Lymphocytes (%)	34.0±8.4	33.3±8.2	33.2±8.6	32.8±8.3	-0.44	0.03
Monocytes (%)	7.0±2.2	6.9±2.3	6.8±2.1	6.8±2.2	-0.18	0.17
Neutrophils (%)	55.6±8.7	56.3±8.9	56.5±9.4	57.0±9.0	0.49	0.01
Eosinophils (%)	3.0±1.9	3.1±2.2	2.9±1.8	3.0±1.9	0.00005	0.99
Basophils (%)	0.51±0.5	0.51±0.5	0.58±0.6	0.63±0.5	0.98	**0.0002**
**MD score**	2.1±1.4	2.3±1.4	2.4±1.4	2.5±1.4	0.70	**0.0001**
**Personal history (P.H.)**						
P.H. of hypercholesterolemia, No. (%)	369 (69.1)	377 (70.6)	362 (67.8)	357 (67.0)	-0.37	0.31
P.H. of hypertriglyceridemia, No. (%)	156 (29.2)	125 (23.4)	149 (27.9)	160 (30.0)	0.12	0.42
P.H. of low HDL, No. (%)	70 (13.1)	72 (13.5)	74 (13.9)	64 (12.0)	-0.18	0.66
P.H. of hypertension, No. (%)	387 (72.5)	384 (71.8)	378 (70.8)	362 (67.9)	-0.26	0.10
P.H. of diabetes, No. (%)	173 (32.4)	154 (28.8)	144 (26.9)	143 (26.8)	-0.34	0.03
**Framingham risk score**	26.3 (16.3; 39.6)	27.3 (16.8; 38.4)	25.6 (16.1; 39.5)	24.5 (16.0; 35.1)	-0.26	0.10
≤5%, No. (%)	3 (0.6)	6 (1.2)	1 (0.2)	3 (0.6)		
>5, ≤10%, No. (%)	41 (8.1)	27 (5.3)	37 (7.2)	37 (7.3)		
>10, ≤15%, No. (%)	67 (13.2)	65 (12.7)	72 (14.0)	78 (15.3)	-0.08	0.33
>15, ≤20%, No. (%)	62 (12.3)	67 (13.1)	75 (14.6)	77 (15.1)		
>20%, No. (%)	333 (65.8)	348 (67.8)	329 (64.0)	315 (61.8)		
**Smoking habits**						
Current smokers, No. (%)	68 (12.7)	77 (14.4)	93 (17.4)	98 (18.4)		
Former smokers, No. (%)	181 (33.9)	214 (40.0)	218 (40.7)	231 (43.3)	0.84	**<.0001**
Never smokers, No. (%)	285 (53.4)	244 (45.6)	224 (41.9)	205 (38.4)		
Duration of smoking (years)†	27 (16; 40)	30 (18; 41)	29 (18; 40)	30 (16; 39)	-0.09	0.64
Cigarettes/day†	15 (10; 20)	15 (10; 20)	15 (10; 20)	15 (10; 20)	0.44	0.85
Pack-years†‡	18 (9; 30)	20 (10; 34)	20 (10; 30)	20 (10; 33)	0.18	0.51
Pack-years$_{code}$						
Never smokers (0), No. (%)	286 (54.8)	244 (46.4)	225 (42.7)	205 (39.2)		
Pack-years (1st tertile), No. (%)	81 (15.5)	78 (14.8)	85 (16.1)	93 (17.8)	0.86	**<.0001**
Pack-years (2nd tertile), No. (%)	73 (14.0)	86 (16.3)	112 (21.3)	101 (19.3)		
Pack-years (3rd tertile), No. (%)	82 (15.7)	118 (22.4)	105 (19.9)	124 (23.7)		
Years since quitting smoking	22 (13; 32)	20 (11; 29)	23 (12; 30)	20 (11; 30)	-0.22	0.27
**Family history (F.H.)**						
F.H. of CHD, No. (%)	329 (64.3)	327 (64.1)	309 (61.1)	321 (62.0)	-0.1	0.30
F.H. of CVD, No. (%)	153 (28.7)	175 (32.7)	183 (34.2)	217 (40.6)	0.78	**<.0001**
F.H. of PVD, No. (%)	57 (10.7)	44 (8.2)	54 (10.1)	58 (10.9)	0.20	0.67
F.H. of hyperlipidemia, No. (%)	195 (36.5)	204 (38.1)	195 (36.4)	232 (43.4)	0.52	0.04
F.H. of hypertension, No. (%)	285 (53.4)	300 (56.1)	299 (55.9)	318 (59.6)	0.35	0.06
F.H. of diabetes, No. (%)	195 (36.5)	186 (34.8)	186 (34.8)	189 (35.4)	-0.08	0.72
**Therapies**						
Statins, No. (%)	199 (37.3)	221 (41.3)	191 (35.7)	221 (41.4)	0.14	0.48
Fibrates, No. (%)	52 (9.7)	35 (6.5)	42 (7.9)	52 (9.7)	0.07	0.81
Fish oil, No. (%)	14 (2.6)	15 (2.8)	26 (4.9)	32 (6.0)	0.55	0.002
Other lipid-lowering drugs, No. (%)	4 (0.7)	1 (0.2)	4 (0.7)	2 (0.4)	-0.23	0.69
Beta-blockers, No. (%)	131 (24.5)	124 (23.2)	125 (23.4)	121 (22.7)	-0.10	0.51
Calcium antagonists, No. (%)	81 (15.2)	90 (16.8)	89 (16.6)	95 (17.8)	0.18	0.29
ACE inhibitors, No. (%)	94 (17.6)	84 (15.7)	119 (22.2)	142 (26.6)	0.97	**<.0001**
Alpha-2 inhibitors (sartans), No. (%)	83 (15.5)	89 (16.6)	68 (12.7)	68 (12.7)	-0.34	0.07
Diuretics, No. (%)	120 (22.5)	128 (23.9)	151 (28.2)	143 (26.8)	0.41	0.04
Anti-platelet agents, No. (%)	103 (19.3)	96 (17.9)	85 (15.9)	90 (16.9)	-0.30	0.20
Insulin, No. (%)	22 (4.1)	25 (4.7)	24 (4.5)	23 (4.3)	-0.06	0.92
Hypoglycaemic drugs, No. (%)	120 (23.0)	104 (19.7)	104 (19.8)	102 (19.4)	-0.28	0.17
Estrogen supplementation, No. (%)	28 (5.2)	29 (5.4)	25 (4.7)	16 (3.0)	-0.40	0.06
**Nutrition variables**						

Wine consumers, No. (%)	203 (38.1)	217 (40.7)	234 (43.8)	251 (47.1)	0.48	0.002
Beer consumers, No. (%)	113 (21.2)	108 (20.3)	81 (15.2)	64 (12.0)	-0.83	**<.0001**
Spirits consumers, No. (%)	98 (18.4)	100 (18.8)	76 (14.2)	59 (11.1)	-0.82	**0.0002**
Fruit consumers, No. (%)	499 (93.5)	503 (94.2)	504 (94.4)	514 (96.4)	0.50	0.04
Milk consumers, No. (%)	427 (80.3)	421 (79.0)	420 (78.8)	410 (77.1)	-0.19	0.22
Coffee consumers, No. (%)	476 (89.5)	485 (90.8)	479 (89.9)	469 (88.0)	-0.28	0.36
Tea consumers, No. (%)	237 (44.6)	194 (36.4)	219 (41.1)	201 (37.7)	-0.15	0.10
Meat consumers, No. (%)	520 (97.9)	525 (98.5)	525 (98.9)	525 (98.5)	0.25	0.39
Fish consumers, No. (%)	487 (91.7)	499 (93.5)	470 (88.5)	475 (89.1)	-0.56	0.02
Eggs consumers, No. (%)	412 (77.6)	414 (77.7)	422 (79.5)	432 (81.1)	0.32	0.12
**Type of fat consumed**						
Butter consumers, No. (%)	32 (6.0)	34 (6.4)	33 (6.2)	19 (3.6)		
Lard consumers, No. (%)	1 (0.2)	0 (0.0)	0 (0.0)	1 (0.2)		
Olive oil consumers, No. (%)	198 (37.1)	217 (40.7)	263 (49.2)	328 (61.5)	-4.44§	**<.0001**
Seed oil consumers, No. (%)	99 (18.5)	74 (13.9)	56 (10.5)	46 (8.6)		
Margarine consumers, No. (%)	202 (37.8)	206 (38.6)	173 (32.3)	135 (25.3)		
**Type of milk consumed**						
Not skimmed milk consumers, No. (%)	15 (3.5)	34 (8.1)	42 (10.0)	33 (8.0)		
Semi-skimmed milk consumers, No. (%)	208 (48.7)	212 (50.4)	234 (55.7)	254 (62.0)	-3.64ǀǀ	**<.0001**
Skimmed milk consumers, No. (%)	204 (47.8)	175 (41.6)	144 (34.3)	123 (30.0)		

Data are n (percentage) or mean±SD, except for triglycerides, uric acid, hs-CRP, blood glucose, creatinine, adiponectin, leucocytes, Framingham risk score, duration of smoking, cigarettes/day, pack-years and years since quitting smoking, which are summarized as median (1st and 3rd quartiles). GSM = gray-scale median. Std. = standardized. MD score = Mediterranean Diet score. CHD = coronary heart disease. CVD = cerebrovascular disease. PVD = peripheral vascular disease. * calculated by using the following latitude values: 62 for Kuopio, 59 for Stockholm, 53 for Groningen, 48 for Paris, 45 for Milan, and 43 for Perugia. † calculated excluding never smokers. ‡ calculated as the number of cigarettes smoked per day multiplied by the number of years smoked/20. § calculated as Not fat consumers Vs. Butter consumers. ǀǀ calculated as Not skimmed milk consumers Vs. Skimmed milk consumers.

To convert biochemical risk factors in mmol/L multiply values of total and HDL cholesterol by 0.0259016, and values of triglycerides by 0.0113815. To convert biochemical risk factors in μmol/L multiply values of uric acid by 59.48. To convert biochemical risk factors in mg/dL divide the values of blood glucose by 0.0556122 and the values of creatinine by 87.777778. Group differences were assessed by ANOVA with Bonferroni's correction for multiple comparisons for the numerical variables, by χ2-test or Fisher test for the categorical ones, and by Kruskal-Wallis for educational level, triglycerides, uric acid, hs-CRP, blood glucose, creatinine, adiponectin, leucocytes, Framingham risk score, duration of smoking, cigarettes/day, pack-years and years since quitting smoking. The p values refer to the trends across carotid plaque-GSM quartiles. P value p<0.0007 (threshold according to Bonferroni correction for 73 comparisons) are in bold.

Table S2. Characteristics of participants according to quartiles of common carotid IM-GSM.

Quartiles of common carotid IM-GSM						
	More echolucent (darkest)			Less echolucent (brightest)		
	(<35.1) (n=797)	(35.1-42.3) (n=797)	(42.3-51.0) (n=797)	(>51.0) (n=797)	Std. Beta coefficients	P_{trend}
Kuopio, No. (%)	283 (35.5)	173 (21.7)	62 (7.8)	15 (1.9)		
Stockholm, No. (%)	139 (17.4)	180 (22.6)	147 (18.4)	66 (8.3)		
Groningen, No. (%)	55 (6.9)	106 (13.3)	124 (15.6)	242 (30.4)	-3.82*	**<.0001**
Paris, No. (%)	233 (29.2)	153 (19.2)	90 (11.3)	25 (3.1)		
Milan, No. (%)	65 (8.2)	112 (14.1)	176 (22.1)	200 (25.1)		
Perugia, No. (%)	22 (2.8)	73 (9.2)	198 (24.8)	249 (31.2)		
**Anthropometric variables**						
Male sex, No. (%)	392 (49.2)	379 (47.6)	319 (40.0)	370 (46.4)	-0.23	0.045
Age (years)	64.9±5.3	64.9±5.4	64.0±5.5	62.9±5.4	-1.73	**<.0001**
Height (m)	1.68±0.1	1.67±0.1	1.66±0.1	1.67±0.1	-0.23	0.009
Weight (kg)	80.2±16.0	76.0±14.2	74.2±15.4	74.8±15.6	-1.23	**<.0001**
BMI (Kg/m^2)	28.3±4.4	27.0±4.1	26.8±4.2	26.7±4.3	-1.44	**<.0001**
Waist (cm)	97.9±13.2	93.8±12.0	92.8±12.7	92.4±12.5	-1.64	**<.0001**
Hip (cm)	103.7±9.7	102.2±9.2	101.7±9.9	102.0±10.1	-0.65	**0.0002**
Waist/hip ratio	0.94±0.1	0.92±0.1	0.91±0.1	0.91±0.1	-1.71	**<.0001**
Heart rate (bpm)	65.2±8.7	66.2±9.3	66.9±9.0	67.4±8.9	0.95	**<.0001**
DBP (mmHg)	82.0±10.9	82.0±10.2	81.6±9.5	82.3±8.9	0.19	0.70
SBP (mmHg)	143.0±19.8	142.4±18.7	140.3±18.1	140.4±17.6	-0.47	**0.0006**
Pulse Pressure (mmHg)	61.1±14.2	60.4±14.5	58.7±13.4	58.1±13.3	-0.76	**<.0001**
**Educational level (study years)**	11.0±3.8	10.7±4.0	10.2±3.9	10.0±4.0	-1.10	**<.0001**
**Physical activity**						
Low, No. (%)	143 (18.2)	158 (20.0)	210 (26.4)	180 (22.6)		
Medium, No. (%)	346 (44.0)	363 (45.8)	352 (44.2)	402 (50.4)	-0.52	**<.0001**
High, No. (%)	297 (37.8)	271 (34.2)	234 (29.4)	215 (27.0)		
**Biochemical risk factors**						
Total Cholesterol (mg/dL)	211.3±45.5	211.3±43.1	218.1±43.8	218.3±42.9	0.55	**0.00006**
HDL Cholesterol (mg/dL)	48.2±14.4	49.3±14.5	49.0±13.9	47.8±13.4	-0.21	0.53
Triglycerides (mg/dL)	121 (86; 178)	114 (83; 166)	117 (83; 167)	118 (84; 172)	0.03	0.09
LDL Cholesterol (mg/dL)	135.2±39.7	135.6±38.3	142.6±38.9	142.7±38.8	0.70	**<.0001**
Uric acid (mg/dL)	5.4 (4.5; 6.2)	5.2 (4.3; 6.0)	5.1 (4.4; 6.0)	5.1 (4.3; 6.0)	-0.63	**0.00002**
hs-CRP (mg/L)	2.1 (0.9; 3.8)	1.8 (0.8; 3.5)	2.0 (0.8; 3.6)	2.0 (0.9; 4.0)	-0.32	0.12
Blood glucose (mmol/L)	5.8 (5.1; 6.8)	5.6 (5.1; 6.4)	5.3 (4.8; 6.1)	5.2 (4.7; 6.0)	-1.96	**<.0001**
Creatinine (μmol/L)	78.6 (68.2; 90.1)	78.9 (67.9; 90.4)	77.0 (66.9; 88.3)	79.9 (68.5; 91.7)	0.26	0.052
Adiponectin (μg/mL)	9.0 (4.8; 15.4)	10.8 (6.2; 18.1)	11.7 (7.3; 18.8)	12.1 (7.4; 19.1)	1.81	**<.0001**
**Hematological variables**						
Leucocytes (WBC) (x 10^9/L) (log)	6.0 (5.2; 7.1)	5.9 (5.1; 7.0)	5.9 (5.0; 6.9)	6.0 (5.2; 7.1)	-0.19	0.11
Erythrocytes (RBC) (x 10^{12}/L)	4.6±0.4	4.6±0.4	4.7±0.4	4.7±0.4	0.60	0.001
Haemoglobin (g/dL)	14.2±1.1	14.1±1.1	14.2±1.2	14.1±1.2	-0.39	0.10
Haematocrit (%)	42.1±3.9	41.8±3.6	42.2±3.2	42.2±4.0	0.16	0.26
MCV (fl)	90.7±4.5	90.5±4.7	90.5±4.5	89.8±5.5	-0.85	**0.0003**
MCH (pg)	30.8±1.6	30.6±1.7	30.4±1.7	30.2±1.8	-1.30	**<.0001**
MCHC (g/dL)	33.9±1.0	33.8±1.0	33.6±1.1	33.6±1.2	-1.06	**<.0001**
Platelets (x 10^9/L)	238.0±57.1	238.8±59.3	239.4±52.9	240.3±59.2	0.15	0.41
Lymphocytes (%)	34.4±8.3	33.1±8.1	33.4±8.6	32.9±8.3	-0.67	0.002
Monocytes (%)	6.7±1.9	6.9±2.3	6.7±2.2	6.9±2.1	0.19	0.65

Neutrophils (%)	55.3±9.1	56.5±8.8	56.6±9.1	56.7±8.8	0.63	0.002
Eosinophils (%)	3.0±1.9	3.1±2.2	3.0±2.0	3.0±1.9	0.01	0.66
Basophils (%)	0.5±0.5	0.5±0.6	0.6±0.5	0.7±0.5	1.82	**<.0001**
**MD score**	2.2±1.4	2.3±1.4	2.6±1.4	2.5±1.4	0.52	**<.0001**
**Personal history (P.H.)**						
P.H. of hypercholesterolemia, No. (%)	553 (69.4)	553 (69.4)	595 (74.7)	537 (67.6)	-0.19	0.99
P.H. of hypertriglyceridemia, No. (%)	245 (30.7)	212 (26.6)	212 (26.6)	240 (30.2)	-0.05	0.82
P.H. of low HDL, No. (%)	109 (13.7)	86 (10.8)	98 (12.3)	101 (12.7)	-0.09	0.79
P.H. of hypertension, No. (%)	571 (71.6)	541 (67.9)	508 (63.7)	506 (63.6)	-0.77	**0.0002**
P.H. of diabetes, No. (%)	250 (31.4)	218 (27.4)	187 (23.5)	183 (23.0)	-0.73	**<.0001**
**Framingham risk score**	25.1 (15.5; 37.9)	23.5 (14.7; 34.0)	20.4 (13.1; 32.4)	21.3 (13.7; 34.3)	-0.74	**<.0001**
≤5%, No. (%)	7 (0.9)	10 (1.3)	10 (1.3)	14 (1.8)		
>5, ≤10%, No. (%)	70 (9.2)	71 (9.3)	93 (12.3)	86 (11.1)		
>10, ≤15%, No. (%)	104 (13.7)	114 (15.0)	137 (18.1)	137 (17.6)	-0.71	**0.0001**
>15, ≤20%, No. (%)	98 (12.9)	122 (16.1)	131 (17.3)	110 (14.1)		
>20%, No. (%)	480 (63.2)	443 (58.3)	388 (51.1)	431 (55.4)		
**Smoking habits**						
Current smokers, No. (%)	95 (11.9)	111 (13.9)	107 (13.4)	137 (17.2)		
Former smokers, No. (%)	306 (38.4)	295 (37.0)	283 (35.5)	309 (38.8)	0.69	**<.0001**
Never smokers, No. (%)	396 (49.7)	391 (49.1)	407 (51.1)	351 (44.0)		
Duration of smoking (years)†	27 (16; 38)	28 (17; 40)	28 (16; 38)	29 (15; 40)	0.09	0.68
Cigarettes/day†	15 (10; 20)	14 (10; 20)	15 (10; 20)	15 (10; 20)	0.22	0.16
Pack-years†‡	17.5 (9.2; 30.0)	19 (8.0; 28.8)	18.6 (9.0; 31.5)	18 (8.2; 29.3)	-0.005	0.72
Pack-years$_{code}$						
Never smokers (0), No. (%)	397 (51.1)	391 (50.0)	408 (51.8)	351 (44.3)		
Pack-years (1st tertile), No. (%)	124 (16.0)	130 (16.6)	110 (14.0)	140 (17.7)	0.65	0.01
Pack-years (2nd tertile), No. (%)	135 (17.4)	130 (16.6)	134 (17.0)	146 (18.4)		
Pack-years (3rd tertile), No. (%)	121 (15.6)	131 (16.8)	136 (17.3)	155 (19.6)		
Years since quitting smoking	21 (11; 30)	21 (12; 30)	21 (12; 29)	21 (10; 30)	0.01	0.99
**Family history (F.H.)**						
F.H. of CHD, No. (%)	502 (65.4)	476 (62.5)	449 (58.2)	443 (57.5)	-0.69	**0.0004**
F.H. of CVD, No. (%)	255 (32.0)	279 (35.0)	305 (38.3)	295 (37.0)	0.52	0.02
F.H. of PVD, No. (%)	75 (9.4)	79 (9.9)	93 (11.7)	84 (10.5)	0.21	0.29
F.H. of hyperlipidemia, No. (%)	324 (40.7)	299 (37.5)	358 (44.9)	365 (45.8)	0.55	0.004
F.H. of hypertension, No. (%)	435 (54.6)	440 (55.2)	454 (57.0)	483 (60.6)	0.48	0.01
F.H. of diabetes, No. (%)	295 (37.0)	294 (36.9)	250 (31.4)	255 (32.0)	-0.32	0.006
**Therapies**						
Statins, No. (%)	298 (37.4)	310 (38.9)	310 (38.9)	309 (38.8)	0.20	0.59
Fibrates, No. (%)	73 (9.2)	65 (8.2)	74 (9.3)	69 (8.7)	-0.18	0.93
Fish oil, No. (%)	13 (1.6)	32 (4.0)	41 (5.1)	39 (4.9)	0.57	**0.0004**
Other lipid-lowering drugs, No. (%)	9 (1.1)	3 (0.4)	5 (0.6)	6 (0.8)	-0.20	0.51
Beta-blockers, No. (%)	203 (25.5)	173 (21.7)	175 (22.0)	157 (19.7)	-0.41	0.01
Calcium antagonists, No. (%)	128 (16.1)	129 (16.2)	103 (12.9)	142 (17.8)	0.21	0.73
ACE inhibitors, No. (%)	126 (15.8)	136 (17.1)	159 (19.9)	184 (23.1)	0.83	**<.0001**
Alpha-2 inhibitors (sartans), No. (%)	165 (20.7)	107 (13.4)	102 (12.8)	72 (9.0)	-1.18	**<.0001**
Diuretics, No. (%)	208 (26.1)	187 (23.5)	188 (23.6)	198 (24.8)	-0.06	0.59
Anti-platelet agents, No. (%)	159 (19.9)	125 (15.7)	104 (13.0)	87 (10.9)	-1.01	**<.0001**
Insulin, No. (%)	38 (4.8)	31 (3.9%)	28 (3.5)	27 (3.4)	-0.23	0.14
Hypoglycaemic drugs, No. (%)	178 (22.8)	155 (19.9)	119 (15.1)	125 (16.0)	-0.78	**<.0001**
Estrogen supplementation, No. (%)	53 (6.6)	45 (5.6)	38 (4.8)	33 (4.1)	-0.53	0.02
**Nutrition variables**						
Wine consumers, No. (%)	310 (38.9)	336 (42.3)	368 (46.2)	369 (46.5)	0.54	0.0007
Beer consumers, No. (%)	158 (19.9)	132 (16.6)	101 (12.7)	79 (10.0)	-1.20	**<.0001**

Spirits consumers, No. (%)	146 (18.3)	111 (14.0)	94 (11.8)	65 (8.2)	-1.25	**<.0001**
Fruit consumers, No. (%)	741 (93.0)	754 (94.7)	758 (95.2)	770 (97.0)	0.84	**0.0003**
Milk consumers, No. (%)	620 (78.0)	629 (79.1)	614 (77.3)	617 (77.9)	-0.04	0.76
Coffee consumers, No. (%)	703 (88.3)	718 (90.4)	697 (87.7)	715 (90.2)	0.14	0.57
Tea consumers, No. (%)	328 (41.3%)	336 (42.3)	274 (34.5)	300 (37.8)	-0.42	0.02
Meat consumers, No. (%)	777 (97.9)	779 (98.1)	782 (98.5)	782 (98.7)	0.23	0.14
Fish consumers, No. (%)	733 (92.2)	727 (91.6)	729 (91.8)	689 (87.0)	-0.88	0.0009
Eggs consumers, No. (%)	618 (77.8)	617 (77.7)	620 (78.1)	648 (81.8)	0.34	0.057
**Type of fat consumed**						
Butter consumers, No. (%)	61 (7.7)	44 (5.5)	33 (4.2)	22 (2.8)		
Lard consumers, No. (%)	0 (0.0)	1 (0.1)	2 (0.3)	0 (0.0)		
Olive oil consumers, No. (%)	290 (36.5)	364 (45.7)	511 (64.3)	513 (64.4)	-11.37§	**<.0001**
Seed oil consumers, No. (%)	135 (17.0)	104 (13.1)	58 (7.3)	67 (8.4)		
Margarine consumers, No. (%)	306 (38.5)	279 (35.0)	186 (23.4)	180 (22.6)		
**Type of milk consumed**						
Not skimmed milk consumers, No. (%)	31 (5.0)	60 (9.5)	46 (7.5)	46 (7.5)		
Semi-skimmed milk consumers, No. (%)	302 (48.7)	329 (52.3)	373 (60.7)	393 (63.7)	-2.32‖	**<.0001**
Skimmed milk consumers, No. (%)	287 (46.3)	240 (38.2)	195 (31.8)	178 (28.8)		

Data are n (percentage) or mean±SD, except for triglycerides, uric acid, hs-CRP, blood glucose, creatinine, adiponectin, leucocytes, Framingham risk score, duration of smoking, cigarettes/day, pack-years and years since quitting smoking, which are summarized as median (1st and 3rd quartiles). GSM = gray-scale median. Std. = standardized. MD score = Mediterranean Diet score. CHD = coronary heart disease. CVD = cerebrovascular disease. PVD = peripheral vascular disease. * calculated by using the following latitude values: 62 for Kuopio, 59 for Stockholm, 53 for Groningen, 48 for Paris, 45 for Milan, and 43 for Perugia. † calculated excluding never smokers. ‡ calculated as the number of cigarettes smoked per day multiplied by the number of years smoked/20. § calculated as Not fat consumers Vs. Butter consumers. ‖ calculated as Not skimmed milk consumers Vs. Skimmed milk consumers.

To convert biochemical risk factors in mmol/L multiply values of total and HDL cholesterol by 0.0259016, and values of triglycerides by 0.0113815. To convert biochemical risk factors in μmol/L multiply values of uric acid by 59.48. To convert biochemical risk factors in mg/dL divide the values of blood glucose by 0.0556122 and the values of creatinine by 87.777778. Group differences were assessed by ANOVA with Bonferroni's correction for multiple comparisons for the numerical variables, by χ2-test or Fisher test for the categorical ones, and by Kruskal-Wallis for educational level, triglycerides, uric acid, hs-CRP, blood glucose, creatinine, adiponectin, leucocytes, Framingham risk score, duration of smoking, cigarettes/day, pack-years and years since quitting smoking. The p values refer to the trends across common carotid IM-GSM quartiles. P value $p<0.0007$ (threshold according to Bonferroni correction for 73 comparisons) are in bold.

Table S3. Cross-validation of independent determinants of carotid plaque-GSM.

	Proportion selected	% selected	Proportion confirmed	% confirmed
Latitude	200/200	100	200/200	100
Diastolic blood pressure	192/200	96	191/192	99.5
IMT_{max} (quartiles)	179/200	89.5	169/179	94.4
Pack-$years_{code}$	152/200	76	127/152	83.6
ACE inhibitors	108/200	54	71/108	65.7
Waist/hip ratio	101/200	50.5	26/101	25.7
F.H. of hyperlipidemia	71/200	35.5	0/71	0
Fibrates	64/200	32	3/64	4.7
MCV	63/200	31.5	6/63	9.5
F.H. of CVD	60/200	30	7/60	11.7
Heart rate	58/200	29	5/58	8.6
LDL Cholesterol	57/200	28.5	7/57	12.3
Creatinine (log)	42/200	21	1/42	2.4
Other lipid lowering drugs	33/200	16.5	0/33	0
F.H. of CHD	29/200	14.5	0/29	0
Total Cholesterol	24/200	12	1/24	4.2
Calcium antagonists	20/200	10	0/20	0
BMI	19/200	9.5	0/19	0
Age	18/200	9	0/18	0
Hypoglycaemic drugs	12/200	6	0/12	0
Statins	12/200	6	0/12	0
P.H. of hypertension	10/200	5	1/10	10
F.H. of hypertension	9/200	4.5	0/9	0
Systolic blood pressure	7/200	3.5	1/7	14.3
Triglycerides (log)	7/200	3.5	0/7	0
Neutrophils	6/200	3	2/6	33.3
Leucocytes (WBC) (log)	6/200	3	0/6	0
Male sex	6/200	3	0/6	0
Beta-blockers	5/200	2.5	0/5	0
F.H. of PVD	5/200	2.5	0/5	0
Fish oil	5/200	2.5	0/5	0
Diuretics	4/200	2	3/4	75

Blood glucose (log)	4/200	2	0/4	0
hs-CRP (log)	3/200	1.5	0/3	0
HDL Cholesterol	3/200	1.5	0/3	0
P.H. of diabetes	2/200	1	0/2	0
Insulin	2/200	1	0/2	0
Platelets	2/200	1	0/2	0
Alpha-2 inhibitors (sartans)	1/200	0.5	0/1	0
Anti-platelet agents	1/200	0.5	0/1	0
Estrogen supplementation	1/200	0.5	0/1	0
Adiponectin (log)	1/200	0.5	0/1	0
Physical activity	1/200	0.5	0/1	0

F.H. = family history. P.H. = personal history. CVD = cerebrovascular disease. CHD = coronary heart disease.

Table S4. Cross-validation of independent determinants of common carotid IM-GSM.

	Proportion selected	% selected	Proportion confirmed	% confirmed
PF CC-IMT$_{mean}$ (quartiles)	200/200	100	200/200	100
Latitude	200/200	100	200/200	100
Waist/hip ratio	193/200	96.5	192/193	99.5
Fibrates	181/200	90.5	171/181	94.5
Creatinine (log)	169/200	84.5	162/169	95.9
Educational level (study years)	167/200	83.5	141/167	84.4
Height	160/200	80	157/160	98.1
MCV	160/200	80	129/160	80.6
Alpha-2 inhibitors (sartans)	149/200	74.5	137/149	91.9
Pack-years$_{code}$	149/200	74.5	137/149	91.9
Systolic blood pressure	141/200	70.5	133/141	94.3
Age	139/200	69.5	104/139	74.8
F.H. of hyperlipidemia	133/200	66.5	77/133	57.9
Diastolic blood pressure	93/200	46.5	69/93	74.2
Uric acid (log)	85/200	42.5	24/85	28.2
BMI	76/200	38	47/76	61.8
Blood glucose (log)	52/200	26	8/52	15.4
Calcium antagonists	46/200	23	0/46	0
Waist	44/200	22	34/44	77.3
Lymphocytes	42/200	21	0/42	0
ACE inhibitors	35/200	17.5	2/35	5.7
Hypoglycaemic drugs	35/200	17.5	2/35	5.7
P.H. of diabetes	33/200	16.5	3/33	9.1
MCHC	21/200	10.5	0/21	0
Erythrocytes (RBC)	20/200	10	0/20	0
Weight	19/200	9.5	6/19	31.6
Total Cholesterol	18/200	9	0/18	0
Male sex	17/200	8.5	7/17	41.2
Neutrophils	15/200	7.5	0/15	0
Hip	14/200	7	12/14	85.7
Other lipid lowering drugs	14/200	7	0/14	0
Anti-platelet agents	13/200	6.5	0/13	0

Heart rate	11/200	5.5	0/11	0
Pulse Pressure	10/200	5	8/10	80
Haematocrit	8/200	4	0/8	0
Statins	8/200	4	0/8	0
MCH	6/200	3	2/6	33.3
Insulin	6/200	3	1/6	16.7
Triglycerides (log)	6/200	3	0/6	0
Diuretics	6/200	3	0/6	0
Haemoglobin	6/200	3	0/6	0
Beta-blockers	4/200	2	3/4	75
F.H. of CHD	4/200	2	1/4	25
P.H. of hypertension	3/200	1.5	1/3	33.3
F.H. of hypertension	3/200	1.5	0/3	0
LDL Cholesterol	3/200	1.5	0/3	0
Platelets	3/200	1.5	0/3	0
F.H. of CVD	2/200	1	2/2	100
Estrogen supplementation	2/200	1	1/2	50
Leucocytes (WBC) (log)	2/200	1	0/2	0
Adiponectin (log)	2/200	1	0/2	0
Fish oil	1/200	0.5	1/1	100
hs-CRP (log)	1/200	0.5	0/1	0
F.H. of diabetes	1/200	0.5	0/1	0
HDL Cholesterol	1/200	0.5	0/1	0
Physical activity	1/200	0.5	0/1	0

F.H. = family history. P.H. = personal history. CHD = coronary heart disease. CVD = cerebrovascular disease.

Table S5. Odds Ratio (95% CI) for a high atherosclerotic burden (IMT_{max} in the upper quartile; i.e., IMT_{max} ≥2.5 mm) according to quartiles of plaque-GSM and IM-GSM.

		IM-GSM (quartiles)			
		**1**	**2**	**3**	**4**
**Plaque-GSM (quartiles)**	**4**	1 (n=29)	0.84 (0.30; 2.33) (n=83)	1.11 (0.43; 2.85) (n=159)	1.50 (0.61; 3.73) (n=263)
	**3**	1.90 (0.72; 4.98) (n=96)	0.85 (0.33; 2.20) (n=150)	1.27 (0.49; 3.28) (n=139)	1.95 (0.77; 4.94) (n=150)
	**2**	2.00 (0.80; 5.03) (n=169)	1.30 (0.51; 3.28) (n=170)	1.84 (0.72; 4.73) (n=123)	2.02 (0.75; 5.45) (n=73)
	**1**	1.96 (0.80; 4.85) (n=264)	1.97 (0.78; 4.98) (n=160)	2.09 (0.78; 5.59) (n=78)	6.06 (1.93; 19.05) (n=32)

The Odds Ratio (95% CI) of IMT_{max} is calculated after data adjustment for latitude, sex, age, educational level, pulse pressure, and pack-$years_{code}$.

Table S6. Multiple regression analysis showing the independent predictors of carotid plaque-GSM forcing adiponectin into the model. Partial R^2 represents the percent variability of the dependent variable explained by each predictor.

	**Standardized Beta**	***p* Value**	**Partial R^2**
Latitude	-3.17	<.0001	8.6%
Diastolic blood pressure	1.35	<.0001	1.7%
IMTmax (quartiles)	-0.96	<.0001	0.8%
Pack-years$_{code}$	0.86	<.0001	0.7%
Adiponectin (log)	0.13	0.56	0.01%
**Whole Model**			**11.9%**

Table S7. Multiple regression analysis showing the independent predictors of common carotid IM-GSM forcing adiponectin into the model. Partial R^2 represents the percent variability of the dependent variable explained by each predictor.

	Standardized Beta	*p* Value	Partial R^2
Latitude	-4.22	<.0001	11.2%
Waist/hip ratio	-1.89	<.0001	1.0%
PF CC-IMT$_{mean}$ (quartiles)	-1.84	<.0001	1.2%
Systolic blood pressure	1.44	<.0001	1.3%
Height	1.51	<.0001	2.2%
Creatinine (log)	0.97	<.0001	0.4%
Fibrates	-0.93	<.0001	0.6%
Pack-years$_{code}$	0.92	<.0001	0.5%
Educational level (study years)	-0.78	<.0001	0.5%
Alpha-2 inhibitors (sartans)	-0.79	<.0001	0.4%
MCV	-0.81	<.0001	0.5%
Adiponectin (log)	0.18	0.42	0.02%
**Whole Model**			**19.8**%

Figure S1

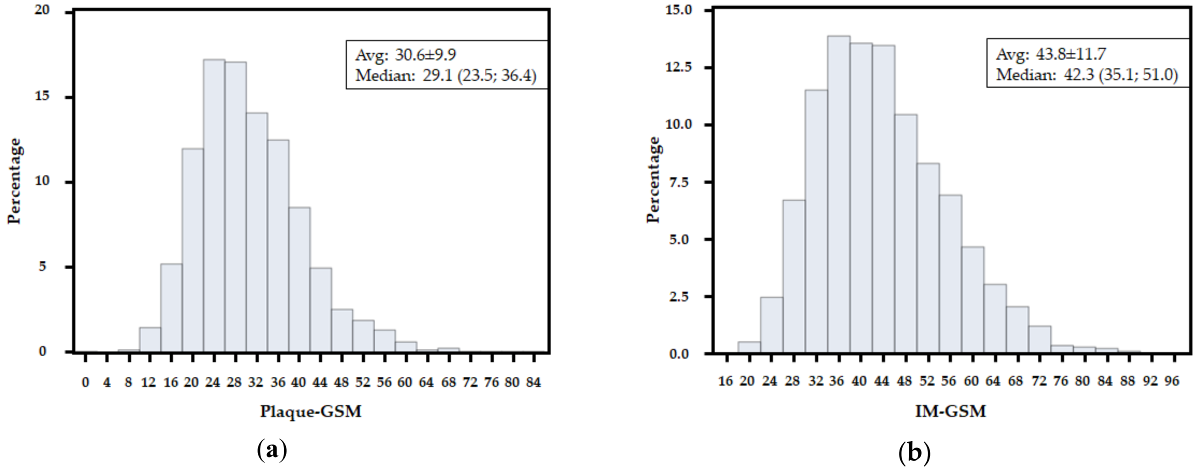

The (**a**) GMS distribution of plaques and (**b**) of IM.

Figure S2

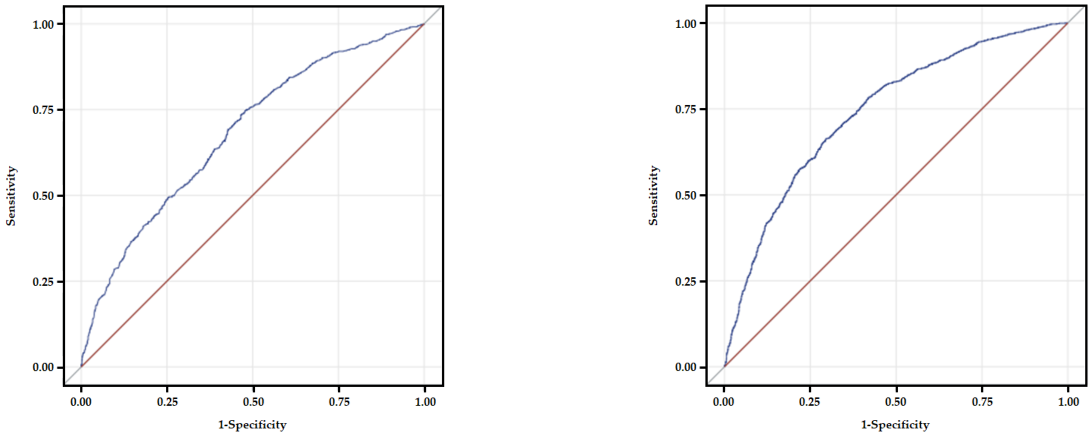

(**a**) (**b**)

ROC curves analyses for (**a**) plaque-GSM and (**b**) IM-GSM. Panel (**a**) adjusted for the independent predictors of plaque-GSM (i.e., latitude, DBP, IMT_{max} (quartiles), pack-$years_{code}$). Panel (**b**) adjusted for the independent predictors of IM-GSM (i.e., latitude, height, SBP, PF CC-IMT_{mean} (quartiles), waist/hip ratio, fibrates, pack-$years_{code}$, MCV, alpha-2 inhibitors (sartans), educational level (study years), and creatinine.

Figure S3

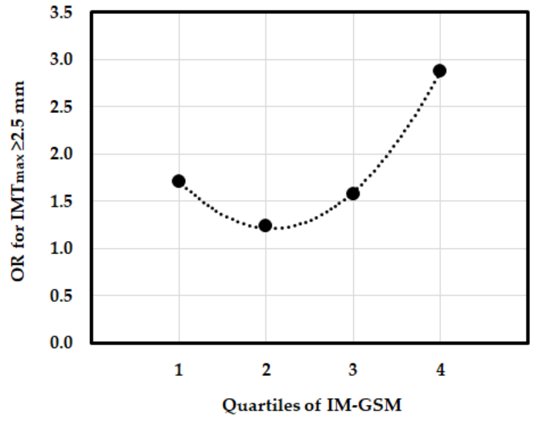

Odds Ratio (OR) for a high atherosclerotic burden (IMT_{max} in the upper quartile, i.e. IMT_{max} ≥2.5 mm) according to quartiles of IM-GSM.

Figure S4

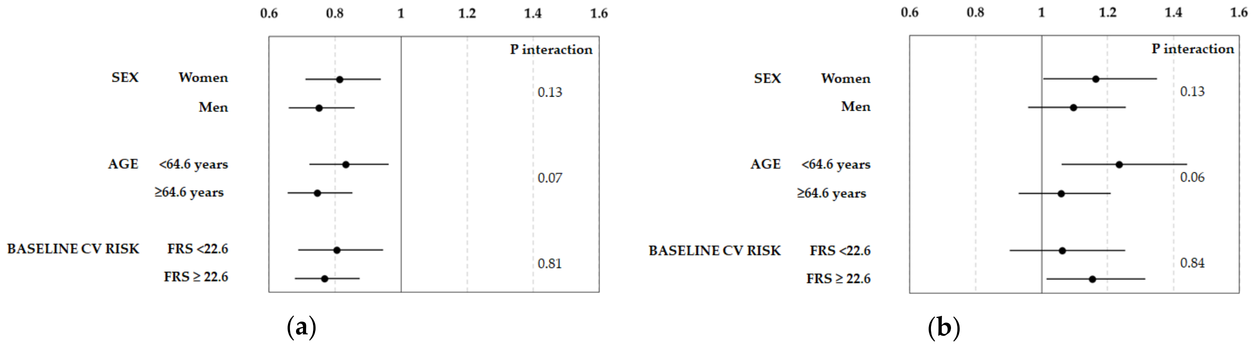

Odds Ratio for high atherosclerotic burden (IMT_{max} ≥2.5 mm), relative to (**a**) a quartile increase of echolucency in plaque (plaque-GSM) or (**b**) a quartile increase of echolucency in plaque-free arterial wall (IM-GSM), stratified by sex, age or baseline cardiovascular risk. OR = odds ratio. CV = cardiovascular. FRS = Framingham Risk Score.

Figure S5

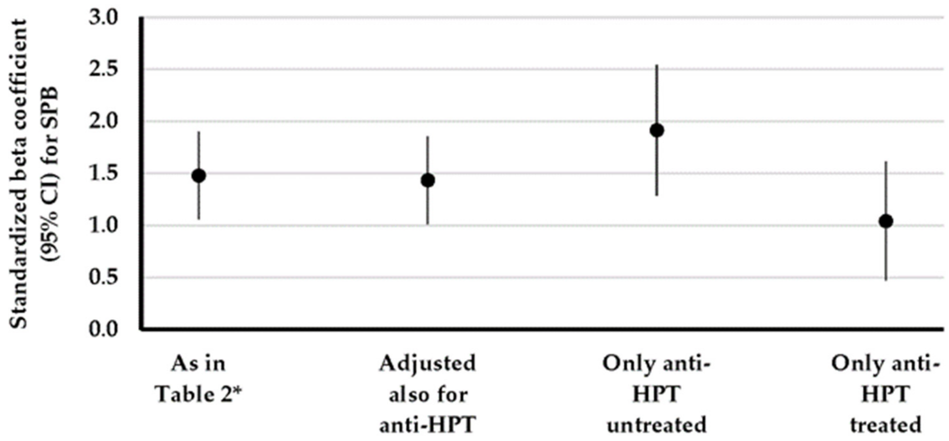

Influence of antihypertensive treatment on the relationship between systolic blood pressure (SBP) and IM-GSM. * Adjusted for PF CC-IMT$_{mean}$ (quartile), latitude, waist/hip ratio, creatinine (log), MCV, height, educational level (study years), fibrates, alpha-2 inhibitors (sartans) and pack-years$_{code}$. SBP × anti-HPT interaction: p=0.056. Anti-HPT = anti-hypertensive treatment.

Figure S6

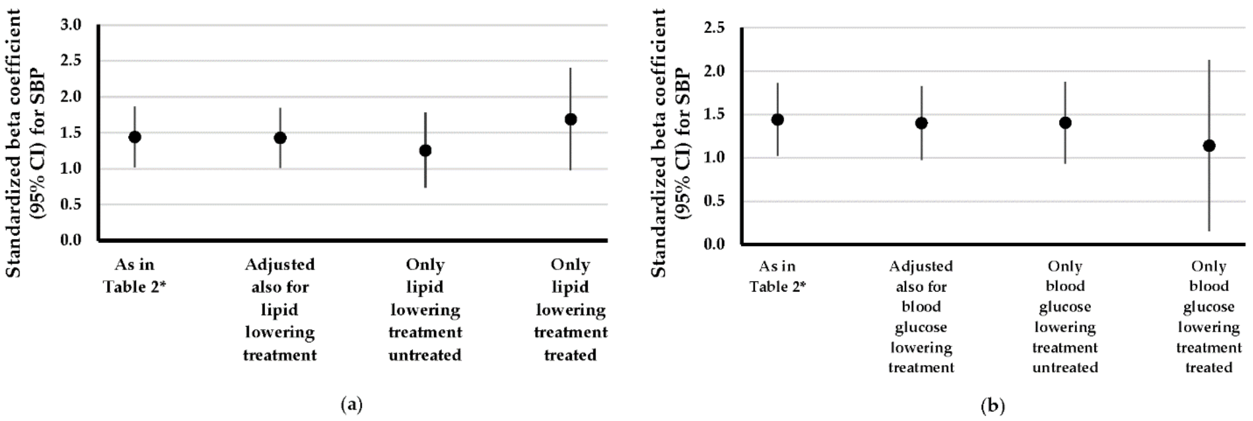

(**a**) Influence of lipid lowering treatment and (**b**) of blood glucose lowering treatment on the relationship between systolic blood pressure (SBP) and IM-GSM. * Adjusted for PF CC-IMT_{mean} (quartile), latitude, waist/hip ratio, creatinine (log), MCV, height, educational level (study years), fibrates, alpha-2 inhibitors (sartans) and pack-$years_{code}$. SBP × lipid lowering treatment interaction: p=0.19; SBP × blood glucose lowering treatment interaction: p=0.43.

Supplementary Appendix

The IMPROVE Study Group

Centro Cardiologico Monzino, IRCCS, Milan, Italy: *Beatrice Frigerio, Daniela Sansaro, Alessio Ravani, Daniela Coggi, Alice Bonomi, Nicolò Capra, Mauro Amato, Damiano Baldassarre.*

Department of Medical Biotechnology and Translational Medicine, Università degli Studi di Milano, Milan, Italy: *Damiano Baldassarre.*

Maria Cecilia Hospital, GVM Care & Research, Cotignola, Ravenna, Italy: *Fabrizio Veglia, Elena Tremoli.*

Dipartimento di Scienze Farmacologiche e Biomolecolari, Università degli Studi di Milano, Milan, Italy: *Laura Calabresi, Cesare R. Sirtori.*

Department of Medicine Solna, Division of Cardiovascular Medicine, Karolinska Institutet, Stockholm, Sweden: *Per Eriksson, Rona J. Strawbridge, Bruna Gigante, Angela Silveira, Anders Hamsten.*

Karolinska University Hospital, Solna, Stockholm, Sweden: *Angela Silveira, Per Eriksson, Anders Hamsten.*

School of Health and Wellbeing, University of Glasgow, Glasgow, United Kingdom: *Rona J. Strawbridge.*

Health Data Research UK, Glasgow, UK.: *Rona J. Strawbridge.*

Division of Cardiovascular and Nutritional Epidemiology, Institute of Environmental Medicine, Karolinska Institutet: *Karin Leander, Federica Laguzzi, Ulf de Faire.*

Cardiovascular Genetics, Institute Cardiovascular Science, University College of London, Rayne Building, University Street, London, United Kingdom: *Steve E. Humphries, Jackie A. Cooper, Jayshree Acharya.*

Foundation for Research in Health Exercise and Nutrition, Kuopio Research Institute of Exercise Medicine, Kuopio, Finland: *Kai Savonen, Kirsi Huttunen, Eva Rauramaa, Ilkka M. Penttila, Jukka Törrönen.*

Department of Clinical Physiology and Nuclear Medicine, Kuopio University Hospital, Kuopio, Finland: *Kai Savonen.*

Department of Medicine, University Medical Center Groningen, Groningen & Isala Clinics Zwolle, Department of Medicine; the Netherlands: *Andries J. Smit, A.I. van Gessel, A.M van Roon, A. Nicolai, D.J. Mulder, G.H. Smeets.*

Assistance Publique - Hopitaux de Paris; Service Endocrinologie-Metabolisme, Groupe Hôspitalier Pitié-Salpetriere, Unités de Prévention Cardiovasculaire, Paris, France*: Philippe Giral, Anatole Kontush, Alain Carrié, Antonio Gallo.*

Internal Medicine, Angiology and Arteriosclerosis Diseases, Department of Medicine and Sugery, University of Perugia, Perugia, Italy*: Matteo Pirro, M.R. Mannarino, G. Vaudo, V. Bianconi, E. Marini, F. Figorilli.*
